# Gold Screws for Retaining Fillings

**Published:** 1876-04

**Authors:** K. B. Davis


### ARTICLE III.

### *Gold Screws for Retaining Fillings.*

BY DR. K. B. DAVIS.

Read before the Illinois State Dental Society.

It is doubtless the desire of every progressive operator to secure greater efficiency in the conservation of the dental organs, by adopting any means, or calling to his aid any methods or appliances that may render his fillings more easy of performance, or that may produce more certain results, than are usually secured, to their integrity and durability.

It is very gratifying that we have an improvement by which we may overcome any danger of fillings loosening or proving failures, in a class of the most difficult cases that we are called upon to treat.

Perhaps in all ordinary cavities little or no difficulty is experienced in securing the filling in such a way as to have it permanently retained; but there are many cases that we are all called upon to treat, not coming under the head of simple cavities, when it is very desirable to resort to something more than the usual methods of anchoring the filling, if we desire the operation to be a permanent one.

Gold screws for retaining fillings were first introduced to the notice of the profession by Dr. Dwinelle, of New York, in the year 1855, and was published in the *American Journal of Dental Science* (Vol. V., new series p. 274.) Perhaps they were occasionally used by various operators, but it seems that they were by no means generally adopted by even the best class of operators. Even at this time, when their merits are so fully and frequently made known by all who are habitually using them, we fear that there are many who have never become acquainted with their value in filling many difficult cavities, where they have hitherto had many failures.

It seems, however, that many practitioners are perfectly satisfied with their present standing as operators, but it is scarcely necessary to remark, that these self-satisfied gentlemen are almost invariably of that class, whose operations are for the most part perfectly worthless, and who are themselves a disgrace to the profession to which they claim to belong. The truly progressive operator is always on the alert for new suggestions, never failing to get out of them all the good they may contain; and no better assurance is needed that a dentist, no matter what his present skill may be, will soon fall behind his fellow-practitioners, than to hear him say that he is perfectly satisfied with his methods of practice, and wishes no better.

The screws and the instruments for their use that are now before the profession, are by no means all that could be desired, and it is to be hoped that some one will soon give us both screws and instruments for their insertion that will be satisfactory.

These little aids in giving our fillings such perfect security are not called into constant requisition like the mallet, rubber dam, and dental engine; but in those particular cases where their use is indicated I do not know of any substitute, nor do I see how any one that wishes to secure the very highest results in conservative practice can dispense with their use. It is true that some operators of high skill and great ingenuity may succeed without their aid, while the masses of the profession, not possessing these happy endowments, would signally fail.

And this brings us to a consideration of the application of screws in filling. I think as a general rule they should be used in every case where we cannot give ample security to the filling by the use of the ordinary retaining pits, grooves, or undercuts. In fact, we can very frequently use them in conjunction with the retaining pits, to give greater stability to our filling, using at eligible points one screw, with one or more retaining pits.

There has been much discussion recently, both in our dental societies and the journals, upon the subject of contour fillings, many urging the impropriety of attempting, as a general rule, to restore the original contour of the teeth in filling; claiming as a reason for this opinion, that such fillings are very much disposed to break off, and thus ultimately result in the loss of the tooth. Now were those who so vehemently argue against contour fillings, in the habit of securing this class of fillings by the use of gold screws, they would readily be convinced of the fact, that by their use these fillings may be made as secure, and retained as permanently, as any other class of simple fillings.

There are two classes of cavities in which the screws are peculiarly indicated. They are, first—all cavities involving the loss of a greater or less portion of the crown, or cutting surfaces of any of the anterior teeth, when we wish to restore the original contour; and in the second place, they should always be used to give greater security to the gold in restoring or building up teeth whose crowns are much worn from the effects of abrasion. Of course, they have a much wider range of application than the above classes of cavities which will readily suggest themselves in each particular case to the experienced operator.

Any one entertaining doubts as to the anchorage secured by the use of screws in contour fillings, will, from the inexorable logic of practical experience, find all such doubts rapidly vanish.

By their use in many instances we are enabled to save a great deal of dental tissue, thus being permitted to fill many cavities without cutting away dentine in order to secure a properly-shaped cavity for the retention of the gold.

Again, they will enable us to save a great many teeth without destroying the pulp. There are many cases where we are called upon when it is impossible to give them a proper retaining shape, without coming into dangerous proximity to the pulp, and in many instances we are compelled to destroy it, owing to a loss of the buccal and lingual walls, in order to get a good foundation for the filling, or otherwise we must resort to the use of the screw, to give security to the filling.

In building up badly abraded incisors or cuspids, I have found the screw invaluable. In many instances these teeth are worn down very nearly to the pulp, necessitating considerable excavating of the only remaining dentine; that is, just above the already much receded pulp, in order to successfully fill them in the old way. Now in order to obviate this, we need only insert from two to four of these little screws into such teeth, without any excavating whatever, and commence our filling; building it up and finishing it with the utmost confidence as to its security and permanent durability.

With reference to the methods of manipulation, it is rather difficult to write,—one observation of their application giving more satisfactory knowledge than many pages of written instructions; and I shall only give, briefly, what I consider to be the chief points to be observed in using them. Then, having a case to treat where we think they are indicated, after having excavated or otherwise prepared the tooth for the operation we select a drill, just the least bit smaller than the screw that we wish to insert, and selecting suitable points in the cavity, or tooth, as the case may be, we make as many pits as we wish for the insertion of the screws. In drilling pits to secure the screws, care should be taken to select a strong wall or portion of dentine so as to give a firm hold to the screws, so that they will be perfectly immovable under the blows of the mallet in consolidating the gold.

I have found from personal experience that the taps belonging to Dr. Mack's set of instruments, and designed to cut a thread in the dentine for the screw are perfectly useless. The screw-threads will cut their own way in the dentine and be very securely held there if we will use a drill as above stated, slightly smaller than the screw, inserting them deep enough to give them a secure footing in the dentine. Having our cavity ready, we take up a screw and with a fine file carefully bevel the square end that is to be inserted into the dentine; we carry it to the retaining point prepared for it, and with a few turns of the driver we secure it firmly enough to hold the filling. It is not necessary to insert all of the screws that we wish to use in one filling at once; we can insert one, and then commence introducing the gold, and then as occasion requires, put in the others.

We will usually have to start the filling by making a small retaining point near one of the screws, and after filling this we will continue to condense the gold around the screws as compactly as possible until they are covered, when the filling is finished in the usual way.

It will be readily perceived that the use of gold screws does not facilitate the operation of filling, as they are very much in the way in packing the gold; nevertheless, we deem it the duty of every operator to avail himself of every facility that will enhance his usefulness as an individual member of the profession, and at the same time promote the highest interest of his patients.

---

